# The link between obesity and migraine in childhood: a systematic review

**DOI:** 10.1186/s13052-017-0344-1

**Published:** 2017-03-07

**Authors:** G. Farello, P. Ferrara, A. Antenucci, C. Basti, A. Verrotti

**Affiliations:** 1 Department of Pediatrics, University of L’Aquila, Via Vetoio 1, Coppito, 67100 L’Aquila, Italy; 2 Department of Pediatrics, Catholic University, A. Gemelli Hospital, Rome, Italy

**Keywords:** Obesity, Headache, Migraine, Children, Proinflammatory cytokines

## Abstract

Obesity and headache are two highly prevalent diseases both in adults and children and they are associated with a strong personal and social impact. Many studies suggest that obesity is comorbid with headache in general, and migraine in particular and obesity seems to be a risk factor for migraine progression and for migraine frequency both in adults and in children. Research shows that there are multiple areas of overlap between migraine pathophysiology and the central and peripheral pathways regulating feeding: inflammatory mediators such as the calcitonin gene-related protein (CGRP), neurotransmitters such as serotonin, peptides such as orexin and adipocytokines such as adiponectin (ADP) and leptin could explain the common pathogenesis. In this paper we discussed the association between obesity and migraine through the analysis of the most recent studies in children and we reviewed data from literature in order to assess the association between obesity and headache and to clarify the possible common pathogenic mechanisms.

## Background

Obesity and headache are two common diseases in adults and children and many data suggest their frequent association [1]. Both are influenced by genetic and environmental risk factors and are related with an increased risk of developing chronic diseases with a marked personally impact and high healthcare costs [2].

Type 2 diabetes mellitus, dyslipidemia, metabolic syndrome, hyperandrogenemia with hyperinsulinemia and consequent polycystic ovary disease, high systolic blood pressure, proteinuria, nonalcoholic fatty liver disease, orthopedic pathologies, pseudotumor cerebri and depression are just some of the conditions associated with obesity [3]. In addition, recent researches suggest a role of obesity on migraine outcomes [1, 4]. In fact, the link between obesity and headache seems to be relatively specific only for migraine [5] and studies in adults and children showed that obesity is a risk factor for migraine progression and for migraine frequency both in adults [6–9] and in children [10–12]. The link between migraine and obesity is multifactorial: inflammatory mediators such as the calcitonin gene-related protein (CGRP), neurotransmitters such as serotonin, peptides such as orexin, and adipocytokines such as adiponectin and leptin play a role both in feeding and migraine physiopathology and could explain the common pathogenesis [13].

Furthermore, a decrease in body weight may lead to a reduction in the incidence and severity of headache and my also represent an important therapeutic strategy [14].

The aim of this review is to discuss the association between obesity and migraine through the analysis of the most recent studies in children and to explore the potential role of weight-loss in the treatment of migraine.

## Obesity: definition and prevalence

World Health Organization defines obesity and overweight as clinical conditions characterized by excessive body weight and associated with increased mortality and morbidity. Body mass index (BMI) is the ratio between body weight (in kg) and height (in m^2^) and is universally used to define the degree of obesity. For adults, a BMI of 25.0 to 29.9 kg/m^2^ is defined as overweight and a BMI of 30 kg/m^2^ or higher is defined as obese [15].

For children and adolescents two worldwide BMI criteria are used: the International Obesity Task Force (IOTF) cutoffs and the World Health Organization (WHO) cutoffs. The WHO system was created using data from the 1997 National Center for Health Statistics (NCHS) (from 1 to 24 years) and defines obesity as a BMI >2 SD from the mean of the WHO reference population for children aged between 5 and 19 years. For children under 5 years, WHO defines obesity as a weight-for-height greater than three standard deviations above the WHO Child Growth Standards median [16].

The IOTF cutoffs are based on six large international sample from Brazil, China, United States, Great Britain, Holland, and Singapore and are an extrapolation of the adult BMI cutoff points for obesity (30 kg/m^2^) [17].

Because pediatric cutoffs vary by age, sex and racial differences in adipose tissue, values of BMI are reported on percentiles tables, that are different, depending on the country. Specifically, according to Italian charts, overweight is defined as a BMI ≥85^th^ percentile for sex and age, and obesity is defined as a BMI ≥95^th^ percentile for sex and age [18].

BMI is an indicator related to total individual mass, but it is well recognized that fat mass, expecially abdominal fat mass, is more strictly associated with an increased risk of several pathological states; so, obesity should be most accurately estimated by demonstration of an increase in abdominal fat mass using direct measurements with CT or MRI imaging, or indirect indexes such as waist circumference and bio-impedance. [19]. However direct measurements are not practical in routine and are too much expensive; among indirect measurements, waist circumference is frequently used in diagnosis of abdominal obesity in pediatric population by reporting the values as percentile calculated on tables representing normal values for age, sex and height [20].

Obesity has reached epidemic proportions representing a really social and health emergency. The prevalence of overweight and obesity is steadily increasing among adolescents and children and it is estimated that approximately 41 million children under the age of five are overweight or obese In addition, about 2–8% of the overall costs for health is linked to obesity and the size of the problem in the USA is double compared to Europe, although the rate of increase in European countries is highest [15].

## Migraine: definition and prevalence

The International Headache Society defines migraine as a primary headache disorder characterized by recurrent headaches that can occur with or without aura. Migraine with aura is characterized by transient focal neurological symptoms that usually precede or sometimes join with the headache. In addition, migraine generally lasts 4–72 h and it can be also associated with a premonitory phase, occurring hours or days before the headache, and with a headache resolution phase. Importantly, the diagnostic criteria used in children are less restrictive than those used in adults: attacks may last 2–72 h and migraine is more often bilateral than in adults; unilateral pain usually emerges in late adolescence and migraine headache is usually frontotemporal [21].

As obesity, headache is very common in childhood. In children under 10 years old, headache prevalence has been reported to be approximately 56% [22] and data suggest that from adolescence to early adulthood the prevalence of headache increases and girls have 53% higher odds of headache than boys [23].

## Migraine and obesity: pathophysiological mechanisms

A direct association between obesity and migraine has not been completely understood but it is likely to be multifactorial, related on both central and peripheral pathways regulating feeding and adipose tissue function, that overlap with pathways implicated in migraine pathophysiology [24], as well as lifestyle and environmental factors [25].

Regulation of feeding is controlled by various brain regions, in particular by hypothalamus and its connections, the so-called melanocortin system. The arcuate nucleus (ARC) releases orexigenic and anorexigenic neuropeptides that trasmitte to paraventricular nucleus (PVN), ventromedial nucleus (VM) and lateral hypothalamus (LH). From LH, other two groups of neurons (orexin neurons, which stimulate feeding, and melanin-concentrating hormone neurons, which inhibit feeding), project to the brainstem nuclei (the nucleus of the tractus solitarius and the dorsomotor nucleus of the vagus) where descending inputs are integrated with peripheral inputs from the gastrointestinal system [26]. Hypothalamus plays a crucial role in migrain pathogenesis too: this is suggested by the presence in migraineurs of predromal and postdromal hypothalamic signs, such as food cravings, mood and sleep disorders [27] and confirmed by the finding of hypothalamic activation during acute migraine attacks [28]. Various neurotransmitters and peptides directed by the hypothalamus in regulation of feeding control have also a role in migraine pathophysiology:

- Serotonin is a neurotransmitter synthesized from the essential aminoacid tryptophan. At the hypothalamic level serotonin directly activate anorexigenic neurons and cause the release of α-melanocyte-stimulating hormone; serotonin is also linked to other hypothalamic peptides involved in feeding control as Neuropeptide-Y and Orexin [29]. Serotonin is a major player in the process of feeding control, signaling the satiety state. This is supported by the finding that serotonin agonist drugs markedly reduce appetite and lead to weight loss. Furthermore, several studies have noted chronically low interictal levels of serotonin in migraineurs [30] and increased levels during acute attacks [31]. Probably serotonin deficit in migraineurs could also explain tendency for increased appetite [32].
- Orexines are hypothalamic peptides involved in several functions including feeding, sleep and hormone secretion, and even in modulation of nociceptive processing [33]. Specifically, Orexin A and Orexin B are secreted by hypothalamic neuronal cells and modulate feeding control: administered centrally in rats they increase food intake [34–36]; orexin A levels seems to be elevated in the cerebrospinal fluid of migraine patients and in chronic daily headache sufferers, probably due to orexin resistance or disruption in orexin receptors [37] and animal and human data support a role for orexins in pain processing [38]. In an animal model, orexin A inhibits neurogenic dural vasodilatation and reduces release of calcitonin gene related peptide (CGRP) from trigeminal neurons [39].
- CGRP is a 37 amino acid neuropeptide which plays an important role in migraine: this is assessed by several studies that found elevated values in the jugular outflow during migraine attacks and normalized values parallel to pain resolution [40, 41]. In another study is demonstrated that CGRP injected intravenously caused a delayed moderate or severe headache in migraine patients but not in non-migraineurs [42]. This evidence supports the hypothesis of CGRP being important in the induction as well as in the perpetuation of the migraine attack: migraineurs may have an increased sensitivity to CGRP due to differences in expression of the functional rate limiting subunit of the CGRP receptor [43]. On the other hand there is strong evidence suggesting that CGRP has immunomodulatory effects and it shows potent anti-inflammatory effects in several animal models of inflammation [44] and in humans, CGRP has been reported elevated in conditions where inflammation is implicated [45]. Since obesity is related to a chronic inflammation too, it is plausible that chronically elevated circulating levels of CGRP, or increased sensitivity to otherwise normal levels of CGRP, may decrease the trigeminovascular activation threshold and increase or trigger frequency of migraine attacks [46].

In addition to peptides and neurotransmitters, adipocytokines participate in energy homeostasis and regulation of feeding; some of them, such as ADP and leptin, play a central role even in immune and inflammatory process and are produced by adipose tissue [24].

Adipose tissue produces both pro and anti-inflammatory mediators that influence local and systemic inflammation [47, 48]. The balance of pro- and anti-inflammatory adipokines is dictated by different factors including nutritional and metabolic status, presence of infection or systemic inflammation, oxidative stress, age and sex [49, 50].

The expansion of subcutaneous adipose tissue hyperplasia mediated is associated with a lower risk of metabolic disease, higher levels of ADP, and lower levels of proinflammatory adipokines. In contrast, the expansion of adipose tissue through the process of hypertrophy is prevalent in visceral adipose tissue and is highly correlated with the accumulation of immune cells, proinflammatory adipokines, and metabolic dysfunction [51, 52]. These molecules are involved in the pathogenesis of migraine too:

- ADP is predominantly produced by adipocytes and is present in the blood circulation as total ADP and includes three multimers: heavy molecular weight (HMW-ADP), middle molecular weight (MMW-ADP) and low molecular weight (LMW-ADP) [53]. ADP and its multimers have both pro- and anti-inflammatory effects: HMW-ADP can activate proinflammatory pattern and is associated with increases in interleukin (IL)-6 levels, whereas LMW-ADP show an anti-inflammatory activity by reducing IL-6 levels [7]. ADP and its multimers correlate with obesity status, showing inverse correlation with BMI, with insulin sensitivity and serum lipids [54] and are present in cerebrospinal fluid in lower concentration than in blood [55]. In the brain, ADP receptors are expressed in the cortex, hypothalamus, brainstem, circumventricular organs, and on the endothelium of the cerebral microvasculature. ADP signaling is mediated by multiple pathways including some implicated in migraine such as nuclear factor kappa beta, AMP-activated protein kinase, and others [56, 57].
  Several studies report increased total ADP levels in migraine subjects compared to controls, after adjusting for waist/hip ratio or BMI [58, 59], and elevated proinflammatory oligomers directly associated with migraine severity; however substantial methodological differences limit firm conclusions, even considering related metabolic factors that may act as mediators or confounders [60].
- Leptin is another adipokine producted by adipose tissue and present in the brain. It is associated with satiety and obese individuals exhibit high circulating levels, suggesting leptin resistance in obesity [61]. Furthermore, leptin is implicated in the modulation of inflammation and pain, but studies evaluating interictal leptin levels in migraineurs are inconclusive, reporting low or elevated or equal levels in migraineurs compared to controls [62]. Two studies evaluating ictal leptin levels in adults and in a pediatric population, reveal the possibility that leptin may be inversely associated with pain severity, with decreased levels during active pain and increased levels interictally [63, 64].

In addition to adipokines, several cytokines have been described in migraineurs: Tumor Necrosis Factor α (TNFα) and IL-6 are increased in ictally episodes of patients with migraine and TNFα is increased in cerebrospinal fluid of chronic daily headache sufferers [65]; furthermore, ADP and leptin have reciprocal relationships with several of these cytokines, suggesting a possible integration of multiple patterns implicated in migraine attacks pathogenesis.

## Pediatric studies evaluating the association between obesity and migraine

Many studies have been performed on adults in order to evaluate the association between obesity and migraine, on the contrary, only few papers suggesting an association between obesity and migraine in the pediatric population have been published (Table 1). The first study, that specifically examined this relationship, was conducted in 2008 by Pinhas-Hamiel et al. In this study were evaluated 273 children and adolescents and was found an association between migraine and obesity according to the evidence that the prevalence of episodic migraine was of 2.5% in normal weight children, of 4.4% in over-weight children and 8.9% in obese children [12].

**Table 1:** Results of pediatric studies evaluating the association between obesity and headache

Reference	Clinical study design	Number of patients	Age (average age or range)	Sex	Sample population	Findings
Pinhas et al. Obesity 2008	Prospective cross-sectional multicenter study	273	13.3 (9–17)	61% F	BMI ≥5^th^ and ≤85^th^ : 116 BMI ≥85th and ≤95^th^:45 BMI ≥ 95^th:^ 112 Total Headache (HA) participants: 39	The OR of headaches was 4-fold increase in girls with BMI ≥95^th^ ( OR 3.93; CI:1.28-12.1). EM was reported in 8.9% of patients with BMI ≥ 95^th^ ; in 4.4% of patients with BMI ≥ 85^th^ ; in 2.5% of patients with BMI ≤ 85^th^
Kinik et al Cephalalgia 2010	Retrospective cross sectional study	124	12.9 (4–17)	62% F	Rel BMI < 110: 80^a^ Rel BMI ≥ 110 and ≤120: 20 Rel BMI ≥120: 22 Episodic Migraine (EM) with aura: 88 EM without aura 36	Patients with a BMI ≥ 120 : 5.3 ± 2.6 HA/month. Patients with a BMI ≥110 and ≤ 120 : 4.4 ± 2.4 HA/mo. Patients with BMI ≤ 110: 3.6 ± 2.2 HA/mo (*P* = 0.018).
Robberstad et al Neurology 2010	Prospective, cross- sectional study	5847	(13–18)	52% F	Overweight and obese^b^: 891 Total HA participants: 1591	The OR of recurrent HA, migraine and Tension Type Headache (TTH) is respectively 40% greater (OR 1.4, CI: 1.2–1.6, *P* < 0.0001), 60% greater (OR 1.6, CI: 1.4–2.2, *P* < 0.0001) and 40% greater (OR 1.4, CI:1.1–1.6, *P* < 0.0001) in overweight and obese compared with normal weight
Ravid et al Headache 2013	Retrospective, cross sectional study	181	10.1 (4–18)	56% F	BMI ≥ 5th – <85th%: 109 BMI ≥ 85th – <95th%: 48 BMI ≥ 95th%: 24	60% of patients with BMI ≥85th– <95th% and 62.5% of patients with BMI ≥ 95th% had migraines compared with 34% of those with BMI ≥ 5th% – <85th%, (*P* = 0 .01). The odds of EM /Chronic Migraine (CM) was over 2 fold greater in those with BMI ≥ 85th – <95th% (OR 2.4, CI: 1.2–4.7) compared with normal-weight. The odds of EM/CM was 3–5 fold greater in females overweight (OR 3.01, CI: 1.24–7.3) or obese (OR 4.93, CI: 1.46–8.61).
Pakalnis and Kring J Child Neurol. 2012	Retrospective, cross-sectional study	925	12.5 (5–17)	58% F	BMI ≥ 5th – <85th%: 610 BMI ≥ 85th – <95th%: 130 BMI ≥ 95th%: 185 Chronic Daily Headache (CDH) : 63 Chronic Migraine (CM): 37 Chronic Tension Type Headache(CTTH) : 22 Unknown: 4	22% (95% CI: 17.1–27.4) of CDH patients had BMI ≥ 95th% compared with unidentified historical norms (16.3%). 26%(95% CI: 16.5–37.6) of those with chronic or CTTH had BMI ≥ 95th% compared with unidentified historical norms (16%).

^a^Rel BMI calculated with following formula: (participants BMI) × 100/(50th percentile BMI for participant’s age and sex)

^b^Based on International Obesity Task Force, 2000, Cole TJ et al. establishing a standard definition for child overweight and obesity worldwide: International survey. BMJ 2000;320:1240–43. Overweight is defined as the BMI with respect to age and sex that correlates to BMI of 25 at 18 y (adult cutoff in widest use for overweight). Obesity is defined as the BMI with respect to age and sex that correlates to BMI of 30 at 18-y-old (adult cut-off in widest use for obesity)

An increase in the frequency of migraine in obese children compared with normal-weight children was also underlined by Kinik et al., who investigated even the influence of obesity on the severity of migraine in 124 children. The number and the duration of attacks, pain severity and associated symptoms were compared between obese, overweight and normal weight patients. The results highlighted no difference in pain severity and migraine duration among groups, but more frequent attacks in obese than in the overweight and normal weight patients were observed, with a positive correlation with BMI [11].

The relationship between migraine and lifestyle factors was examined by Robberstad et al. in a group of 5847 adolescents. Results highlighted that the risk of migraine was 60% greater in those adolescents who were overweight or obese. The authors suggested that the intervention on lifestyle factors could be a therapeutic strategy [66].

These data were confirmed by Ravid et al., who showed a higher prevalence of obesity, compared with the general population, in a group of 181 children with headaches. This paper evaluated the association between obesity and generally headache and also the association between obesity and migraine specifically. The results showed that migraine was more prevalent in obese and overweight patients compared with normal weight patients. Therefore the odds of migraine were almost 2,5 fold greater in those who were overweight compared with those of normal weight and 3–5 fold greater in overweight and obese females [67].

In 2012 Pakalnis et al., who retrospectively evaluated 925 children with headache, did not show increased incidence of overweight in children with chronic migraine. Specifically no significant differences were noted in the percentage of obese patients with episodic migraine or obese with chronic migraine compared with data from general obese population [68].

## Conclusions

Pediatric studies analyzed in this review confirm that migraine and obesity represent two major public health problems and research is consistent in supporting higher frequency and severity of headache attacks among obese children with migraine than in lean controls.

Adult studies show an associations between obesity and migraine, influenced by age, gender, and lifestyle factors; in pediatric population there is a recurring correlation too, relatively to the increased frequency and intensity of migraine attacks in both sexes, and increased headache episodes in girls.

Studies evaluating the prevalence of overweight in migraineurs children underline a higher prevalence of obesity in these subjects than in the general population.

However, the link between migraine and obesity is complex and still unclear, as the two conditions share some pathogenic determinants and may influence one another.

The two chronic diseases show a multifactorial etiology: genetic factors, inflammatory mediators and common lifestyle factors. A dysregulation of the hypothalamic neuropeptides involved in migraine can induce an alteration in feeding regulation. On the other hand, adipokines producted by hypertrophic adipose tissue create a systemic chronic inflammation that may contribute to the neurogenic inflammation of migraine: this can explain the higher frequency and severity of migraine attacks in overweight and obese children compared to lean controls.

Obesity and its related complications, such as insulin-resistance, are risk factors for chronic migraine: this is highlighted by the evidence that an improvement of metabolic control in obese subjects, through BMI correction, may reduce frequency of migraine attacks.

In conclusion, the link between obesity and migraine is complex and involves both central and peripheral pathways, but obesity and migraine are also both influenced by sedentary lifestyle factors.

These evidences have important clinical implications: body weight management could be relevant for prevention of chronic migraine and represents an alternative treatment option.

In sight of this, it is crucial, in pediatric migraineurs obese patients treat these children precociously with a behavioral therapy targeted to restore the ideal body weight, in order to reduce the frequency of migraine attacks, the use of analgesic therapies and the risk of progression to chronic migraine or chronic daily headache.
